# Fighting adult illiteracy with the help of the environmental print material

**DOI:** 10.1371/journal.pone.0201902

**Published:** 2018-08-23

**Authors:** Tassawar Iqbal, Saqib Iqbal, Syed Sajid Hussain, Iftikhar Ahmed Khan, Hikmat Ullah Khan, Attiqa Rehman

**Affiliations:** 1 Department of Computer Science, COMSATS University Islamabad, Wah Campus, Pakistan; 2 Department of Software Engineering & Computer Science, Al Ain University of Science & Technology, College of Engineering, Al Ain, UAE; 3 Department of Computer Science, COMSATS University Islamabad, Abbottabad Campus, Pakistan; Hangzhou Normal University, CHINA

## Abstract

Adult illiteracy is a major problem worldwide especially in developing countries. Adult Basic Education (ABE) programs working in this context are not very effective due to lack of motivation for the people who are not literate. The reason is inadequate learning content and content delivery methods. This situation calls for developing novel learning content and a learner-directed content delivery approach. This paper presents an exploratory study investigating the use of the Environmental Print Material (EPM) as learning content for the non-literate population of Pakistan. The EPM content is presented to the adult non-literate population in two ethnographic studies. The most frequently recognized content is selected and utilized as learning content in a Computer Assisted Learning (CAL) application. An empirical study is conducted upon two groups with 107 participants to compare the EPM-based learning content with Traditional Learning Content (TLC). As many as 54 participants participated in the experimental group (presented with EPM-based learning content), whereas 53 participants took part in the control group (presented with TLC content). The results reveal that the experimental group performed significantly better compared to the control group in recognition, pronunciation, and recall of the presented content. The meta-analysis of the results shows a large effect size of (1.05) with confidence interval in the range (0.798–1.315). The results claim that the EPM has potential to be considered as learning content in the ABE programs.

## Introduction

Cohen et al. (2015) argued that to achieve sustainability, literacy relevant to sustainability is important [1]. The fundamental aspect of the sustainability literacy is to get everyone literate. There are 774 million non-literates globally, and majority reside in South Asia [2]. Around 58% of the Pakistani population is non-literate, and most of them live in rural areas [3]. Therefore, illiteracy is a major problem of Pakistan. The causes of illiteracy in Pakistan are poverty, high population growth, lack of resources and inconsistent literacy policies [4]. Adult Basic Education (ABE) programs give a solution for the mass eradication of illiteracy. However, a common pitfall of ABE programs is the reduced motivation of the learners. The reasons for this may be poor learning content and inadequate delivery methods [5, 6]. Thus, effective design and delivery of the learning content is a challenge. There have been many efforts for effective design and delivery of learning content. For example, visual cues, such as images, have been found very effective for adult learners as compared to textual content [7, 8]. Aanstoos provided a broader perspective on visual imagery for learning [9]. He found that a "visually literate" person can use the "visual competencies" for interpreting the objects and symbols. Recent research has used such visual imagery for the non-literate population. It has been proved that the audio-visual learning content and multi-modal scaffolding for content delivery improve the motivation of the adult learners [10]. Other researchers have also reported positive outcomes of visual learning content [11, 12].

It is imperative to note that the learning content needs to be relevant to the learners’ background, culture, interests, and preferences [13–15]. Designing the learning content as tasks can enhance interest in learning [16]. The learners must be able to relate to these tasks from their experience. In this context, [13] used Environment Print Material (EPM) as learning content for the non-literate learners. The EPM consists of signs, logos, advertisements, and inscriptions prevalent in the learners’ environment [17]. Adult non-literates, living in a print-rich environment, use their visual competencies to build a sight vocabulary of the EPM. An ABE program can use this sight vocabulary. There has been extensive research on using EPM in emergent literacy programs for children [18]. The potential niche of using EPM for ABE programs has also been explored. For example, Kurvers and colleagues compared the print awareness of non-literates with pre-reading children and low-educated adults in the Netherlands [17]. They reported that the non-literates behaved like children in terms of recognizing EPM. The non-literates could see what the written language looks like in the environmental print but they were unable to recognize the EPM out of context. Some comparable results are reported by [19]. These results show that the exposure of non-literates to the EPM does not automatically guarantee its recognition. This calls for careful planning for using EPM for an ABE program.

The focus of this paper is to select the right learning content based on EPM and to test the effectiveness of this content in teaching adult non-literate population. The adult non-literate population has limited reading skills and almost no writing skills [20]. Therefore, this paper posits the following main (Hm) and sub-hypotheses:

H_m_: The learning content based on EPM can enhance the learning of the adult non-literate population compared to traditional content.
H_1_: The learning content based on EPM can increase the recognition rate of the adult non-literate population.
H_2_: The learning content based on EPM can increase the pronunciation accuracy of the adult non-literate population.
H_3_: The learning content based on EPM can increase the recall (remembrance) rate of the adult non-literate population.

The rest of this paper is organized as follows: Section 2 discusses the state of the art of the design of learning content and their delivery mechanisms to the adult non-literate population. Section 3 presents two short studies conducted to select the EPM, used for designing the learning content. The effect of this learning content and their presentation is studied in an experiment with the adult non-literate population. This experiment is presented in section 4, while the results are presented in section 5. This paper is concluded by section 6 by presenting the conclusions, limitations, and future work.

## State of the art

UNESCO defines non-literates as “the population aged 15 years and above who cannot both read and write with understanding a short simple statement on their everyday life” [21]. The research distinguishes between non-literates from functional illiterates, who have limited reading skills and almost no writing skills [20]. According to Wagner, Information and Communication Technology (ICT) has a potential in implementing ABE programs [22]. The following are some of the reasons for this [23]:

1. An ICT solution has an ability to reach geographically dispersed communities.
2. ICT can give interactive, informal, and time flexible learning platforms for those who are unable to regularly attend formal educational systems.
3. ICT can give individual and customized solutions based on language, gender, and ethnicity etc.
4. A network of educational experts can be formed to support an ICT-based ABE program.

There is a growing body of work for ICT-based literacy solutions for non-literate population [22, 24–28]. The purpose of an ABE program is to give minimal educational skills such as basic reading, writing, and computational skills. This section presents a detailed discussion of the literature about two factors: namely 1) designing the learning content and 2) delivering the learning content in the ABE programs. The aim of this discussion is to name the guidelines proposed in the literature to design and deliver the learning content to the non-literates.

### The design of the learning content

ABE programs have been implemented for the last six decades in Asia. UNESCO has developed best practices for curriculum design defining three levels of literacy skills: basic, middle, and self-learning [29]. The basic level literacy includes the following skills:

1. reading the headlines and sub-headings of newspapers, posters, and numbers from 1–1000,
2. writing one’s own name, simple letters, and numbers from 1–1000,
3. counting and recognizing letter 1–1000, 3-digit addition, and subtraction and
4. other skills such as communication and use of literacy skills in daily life.

The above skills can be acquired incrementally, i.e., reading is acquired first followed by writing counting and communication [29]. Several approaches have been proposed to teach reading. One approach, called the phonics approach, involves teaching the alphabet letters first [30]. Another approach, called the whole language approach, teaches words for building vocabulary using sight experience [14, 31]. The effectiveness of these approaches depends on the interest of adult learners in the learning content [14]. The material in the environment of the learners could increase their interest and thus should be considered while designing learning content.

Designing learning content is a complex job, especially in case of adult learners, who have variant literacy skills. Poorly designed content badly affects the teaching method and hinder the learning process [32]. There are no standards to design learning content for adult learners. However, various guidelines have been proposed to teach adult learners. The following paragraphs discuss these guidelines.

The first guideline is the use of multimodal learning content [10]. It is a combination of textual, visual, audio, gestural, and spatial modes [33]. Multimodal learning content increases the motivation of non-literates [10] and their level of comprehension [34].

The second guideline is to build on the earlier knowledge and to use the past experiences [35]. The learning process adds the latest information to the current structure of knowledge, understanding, and skills of a learner [36]. Prior knowledge of a learner plays a critical role in learning according to constructivism [37]. Learners find the new information helpful if it is presented in a way that complies with the previously developed schemas. This is especially true in case of non-literate learners because they have already developed a number of schemas based on their experiences and exposure to the environment. As problem-oriented learning is helpful for adult learners [35], thus, the learning content must be related to the problems faced by the adult learners in real-life. Also, this content may be related to the past experiences of adult learners to have personalized learning content. Such learning content may motivate the learners and may positively influence their learning experience.

The third major guideline is to contextualize learning. Designing the learning content may vary across geographical regions and cultures [29]. Therefore, contextualized learning informed by the learners’ culture, prior knowledge, and past experiences is also important to the content design. Laves and Wenger found that “learning is embedded within an activity, context, and culture” [16] called as a situated learning theory. This theory states that the knowledge should be presented in a valid context. This contrasts with the classroom learning where the knowledge is delivered out of context. Sociocultural settings of learners are also relevant for situated learning [37]. The situated learning theory has three primary features, the culture of the learners, their context, and authentic tasks. These are the tasks where the learners can relate their experiences and context [38]. When the learning process entails authentic task, it is more likely that the learners would pay more attention and their interest would lead towards engagement and hence they would be more motivated towards the learning [37].

Table 1 presents a summary of the guidelines about designing the learning content proposed in the literature. It lists the features of learning content, their description and their effects on the adult learners.

**Table 1:** Features for designing the learning content for non-literates.

Features	Description	Effects
Multimodality	Combination of textual, visual, audio, gestural and spatial modes [33]	Increases the motivation of non-literates [10] Increases the level of comprehension [34]
Building on the previous knowledge and utilizing the past experiences [35]	Creation of new knowledge schemas and updating of existing schemas	The following effects are reported in the literature [35, 36] Adds information to the current structure of knowledge Increases understanding Increase skills Motivates the learners Affects the learning experience positively
Contextualized learning	Learning is embedded within [16] activity, context and culture The relevance of the learning content to the sociocultural settings of the learners [37]	Learners can relate to their experiences and context Learners pay more attention resulting better engagement and more motivation toward learning

### Delivery of the learning content

Delivering the learning content to non-literates usually follows a non-traditional approach, termed as facilitated learning. In this approach, the role of the instructor is limited to orientation and on-demand help. Knowles observers that adult learners prefer to learn independently [35]. The motivation of adult learners in an ABE program is important. Keller proposed a motivational design model for promoting and sustaining motivation in the learning process. The key constructs of this model are attention, relevance, confidence, and satisfaction [39]. Keller proposed different activities which may help in the enactment of his motivational design model. These activities include a) considering individuals learning styles while designing the learning content, b) supporting active participation, c) learners’ control, and d) giving feedback to learners while delivering the content. All these activities are well-proven in the research on ABE. The following is a brief treatment of these activities considering the contemporary research.

1. The use of learning styles in educational design is explored by various other researchers [40–42].
2. The concept of active participation is in line with Kolb’s theory of experimental design. He writes that “learning is the process whereby knowledge is created through the transformation of experience” [43]. Experimental design can be realized by role activity-based participation in the learning process.
3. Learners’ control, which is also called self-paced learning, offers independent learning opportunities to the learners where they can make their own decisions on browsing the learning material.
4. Learner’s feedback is a critical feature of content delivery. It is important to give feedback to learners to increase learners’ satisfaction, especially in the facilitated learning approach. A manifestation of the learner feedback may be a reward mechanism [39].

While the preceding features of content delivery focus on improving the learning process, the behavior of the learners is also important. The contemporary literature has studied various features of content delivery, which induce positive behavior of adult learners. These features include repetition of content, frequent feedback, and positive reinforcement. Repetition of content is an important feature, which may help to reduce the cognitive load of the learners and offer them flexible learning environment [44]. Drill and Practice software is a software that affords a repetition of learning content [45]. Further, immediate and frequent feedback may help learners to learn from their mistake. Feedback may induce positive reinforcement in learning. According to Skinner, reward and punishment control most of the human behaviors [46]. A reward for a good act is called positive reinforcement and a punishment for an immoral act is called negative reinforcement. The Drill and Practice software supports positive reinforcement through repetitive practice [45].

Table 2 summarizes the above discussion about delivering the learning content to the non-literates in an ABE program. It lists key features of content delivery showed in the preceding discussion and details their effects on the learners.

**Table 2:** Features for content delivery to non-literates.

Features	Description	Effects
Facilitated learning	Instructor’s role is limited to orientation and on-demand help[35].	Considering the learning styles, active participation, learners control and frequent feedback while delivering content promotes and sustains the motivation of the learners [39].
Repetition of content	The learning content is readily available to the learners in multiple modes like visuals, descriptive and audio. This content can be utilized by the learners according to their ease repetitively.	Reduced cognitive load [44] Flexible learning environment [44]
Frequent feedback and positive reinforcement	The learners receive frequent feedback on their learning performance. This feedback is often coupled with rewards in order to have positive reinforcement.	Immediate and frequent feedback may help learners to learn from their mistakes. Reward and punishment control most of the human behaviors [46]. Rewards may induce positive reinforcement, resulting in a longer retention of learning content.

## Ethnographic studies to select the EPM

According to some researchers, learning content can be reused from mainstream education [47, 48]. Although borrowing content from mainstream education is an easy and simple approach, it has many limitations. The approach excludes the importance of learners’ experience, their interest, and motivational aspects [48]. Prevedel reviews the one-size-fits-all approach of using existing content and proposes a learner-driven approach to content designing [48]. The learner-driven approach empowers the learners by considering their needs, interests, their social, and cultural context. However, designing learning content with learner-driven approach gives new research challenges such as:

1. finding the learners’ need and interests, and creation of content considering learners’ needs, and real-life problems, and
2. exploiting commercially available material in this regard [49].

Realizing the potential of the learner-driven approach, various researchers explored it and gave solutions to the challenges. Eberle and colleagues conducted an ethnographic study with the participants of an ABE program to find their needs and interests and used these insights to design the curriculum. They found that the curriculum designed in this way had a positive impact on the learning process [13].

Since the focus of this paper is to design and evaluate the learning content for adult learners belonging to Pakistan, we followed the learner-driven approach. Following the tradition of Eberle et. al. [13], we have used ethnography to design the learning content relevant to the needs and interests of adult learners. This also addressed our first challenge. The second challenge is addressed here by using the EPM as learning content. The rest of this section is divided into two subsections. Section 3.1 highlights the importance of EPM and its role in designing learning content, while section 3.2 presents two ethnographic studies to select the EPM relevant to the cultural context of Pakistani adult learners.

### EPM and the learning content

EPM is the printed material present in our immediate surroundings and used by us in our everyday lives [50]. The examples of EPM include logos, inscriptions, brand names, etc. The research in EPM awareness started in the 1960s and 1970s, and the potential of EPM for literacy programs was first explored in mid-1980s [51]. Most related studies focus on reading skills’ development in children. Kassow gave a literature review of the research on EPM. He reported that the context plays a significant role in reading EPM, and children cannot read an EPM out of context [50]. The reason for this effect is that the children can use contextual cues to recognize the EPM, while out of context EPM presentation results in the recall of the information.

Kurvers and colleagues did seminal work on studying EPM awareness in non-literate adults [17]. They investigated the knowledge of environmental print for children, non-literates, and low-educated subjects, and found that for all groups EPM was recognized better when it is presented in context. Kurvers et al. found two distinct models of learning to read in literature, i.e., a stage model, which involves “explicit and systematic teaching”, and a non-stage model that involves learning through enough exposure to written language [14, 31]. According to the stage model, the names and sounds of letters must be learned first because this knowledge is prerequisite in learning to pronounce words [52]. However, Kurvers et al. argue that the exposure of non-literates to EPM may help them in building sight vocabularies, which may be beneficial in learning to read following a non-stage model. Although research studies on EPM for literacy could rarely recognize the strong association between EPM and developing reading skills (see, e.g., [19]), however, Kurvers et al. found a weak relationship between the two. Moreover, it is a fact that children are capable to recognize environmental print before they can read prints in books [53, 54]. Thus, the literature discussed in this subsection shows that since non-literates have enough exposure to EPM, any learning content based on EPM may augment their learning experience.

### Departmental approval and ethical considerations

In this research, three studies were planned: two ethnographic studies on semi-literate and non-literate people and one experimental study on non-literate people. The studies and experiment were reviewed and approved by the Departmental Ethics Committee—called Project Research & Evaluation Committee (PREC). The consent forms for each one of the studies and the experiment were obtained from the participants respectively. The consent of the parents was obtained for the participants under age of 18 years as approved by the PREC. Since the participants were non-literate and semi-literate, consent was read to them and their verbal approval was taken and recorded in a spreadsheet. The procedure of taking consent in this way was approved by PREC. Only upon the agreement of the participants, the experiment was conducted.

### Ethnographic studies

To study the potential use of EPM as learning content for non-literates, the first step was to select EPM-based content. Since the EPM content is pre-designed and is present in the surroundings, the goal was to select the right EPM content and then to map its characteristics with the features of learning content proposed in the literature (see Table 1). For the purpose, two short ethnographic studies were conducted. The study-I was conducted to collect the often seen EPM, whereas the Study-II was conducted to select the most occurring EPM content, which is easily recognizable for the non-literate population. Table 3 summarizes the purpose, participants, and materials used in the ethnographic studies. The details of the study-I are presented in section 3.3.1, and the details of the Study-II are presented in section 3.3.2.

**Table 3:** Purpose, participants, and materials used in the ethnographic studies.

Study	Purpose	Participants	Materials
Study-I	to collect the EPM often seen by the people with low literacy. The reason is to maximize the number of EPM content available for the selection.	77 participants having some literacy (from level 1 to 4), who could read and write both Urdu and English alphabet letters	Eight EPM content of each Urdu alphabet letter were collected and five most common EPM content were selected
Study-II	To select one EPM content for each Urdu alphabet letters. The reason for selecting these participants was to provide a close match with the participants of the experiment (see section 4)	95 different participants (not participating in the Study-I) having no literacy	One EPM content most often recognized by the participants was selected for each Urdu alphabet letter and it was used as EPM-based Learning Content (ELC) in the experiment (see S1 Table)

#### Study-I

This ethnographic study was conducted in a small town in Pakistan. The participants of this study had the basic ability of reading (alphabet letters and some words) and writing (alphabet letters and some kindergarten-level words) in both English and Urdu. They had between 1–4 years of schooling. The participants could recognize and read the EPM in their surroundings. A total of 77 participants (41 males and 36 females) took part in this study. Their mean age was 30 years within the range 15–55. They practiced varying professions like shopkeeper, laborer, auto-mechanic, security guard and painter etc. The rationale for the choice of such participants for this study was their similarity with non-literate people because they were not true literates. Therefore, their selected material might also be comprehensible to non-literates.

**Fig 1:**
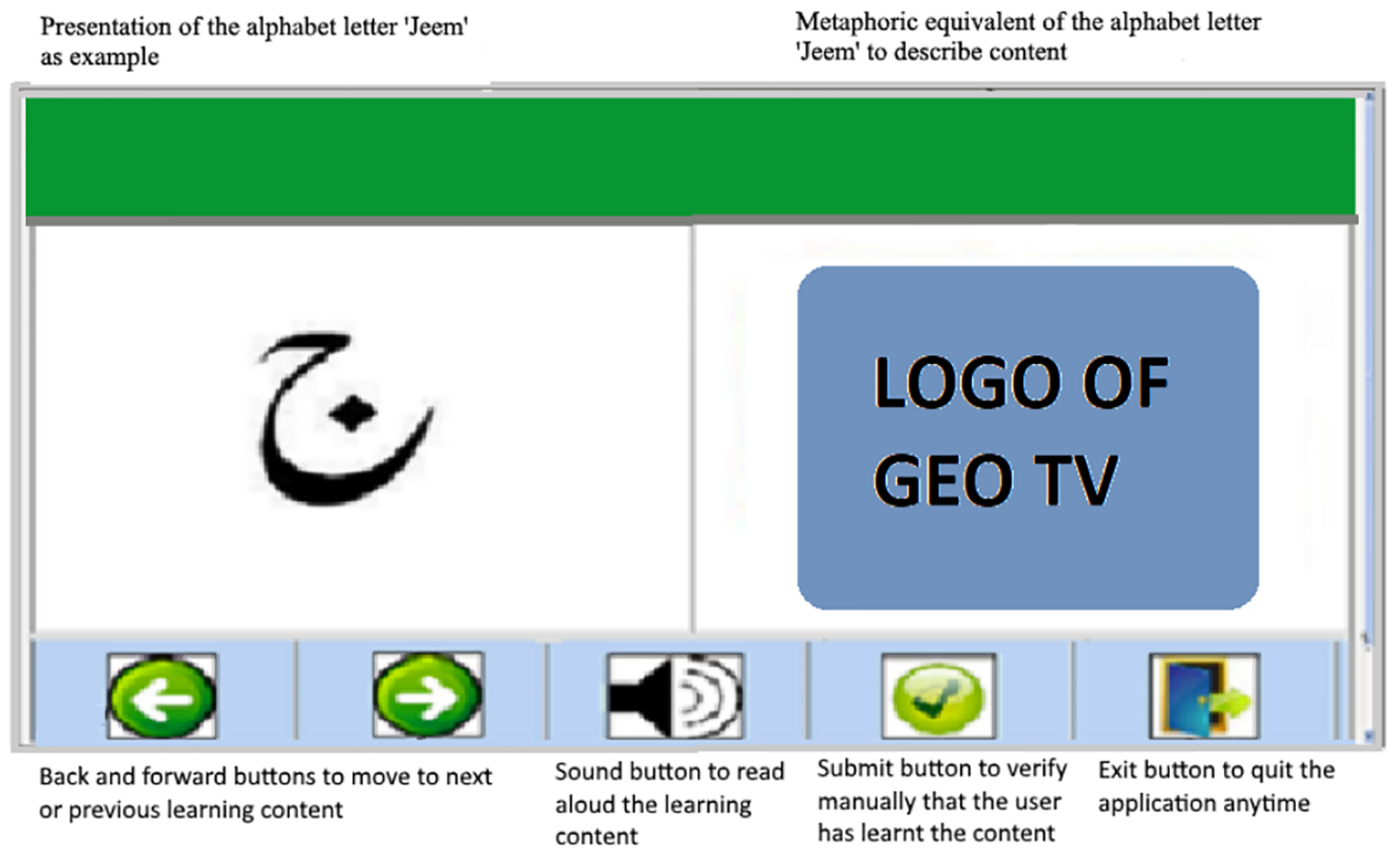
Computer Assisted Learning application presenting the EPM-based learning content (ELC). The traditional content was presented without displaying the EPM content.

A form was designed to collect the EPM content against 28 most often used Urdu alphabet letters. The participants were instructed to write at least eight common content from the EPM whose name starts with the given alphabet letter. The five most often written content for each alphabet letter was selected. After removing all the redundant content, a collection of 140 EPM content remained. This content was categorized as products of daily use, usual places, and known brand names that offer assorted products and services. Fig 1 shows the alphabet letter ‘Jeem’ and the logo of a famous TV news channel ‘GEO’.

#### Study-II

To confirm the selected EPM content, the participants of this study were presented with the content selected from the study-I and were told to recognize it. A total of 95 participants took part in this ethnographic study. Out of 95, 64 were males and 31 were females. Their mean age was 35 and in the range of 17–60. They practiced varying professions as the participants of the earlier study. The graphene-based interviews were conducted to make sure that the participants could recognize and pronounce alphabet letters.

Pictures of the selected EPM content were presented to the participants and they were asked to recognize the EPM content against each alphabet letter. No help was provided to the participants in the recognition process. For each alphabet letter, most often recognized content was selected from the already reduced content. The finally selected EPM content are presented in S1 Table. TLC is the content borrowed from mainstream education [55–57] and contained both alphabet letters and metaphors (see S2 Table). However, metaphors used in TLC were the pictures as used in the mainstream education. This content, therefore, is more suitable for children whereas has less fascination for the adult learners. For example, pictures of animals, fruits, and places. Although, these metaphors have cultural compliance one may have rare interaction with most of the objects used in the metaphors (see S2 Table). Whereas in case of EPM based content one may have frequent interaction in daily life. For instance, the brand metaphors such as Pepsi, Daalda, Zong, Surf, Lipton, Geo, Warid, Ufone, one may use in daily routine and may interact numerous times in a day. Similarly, numerous metaphors such as Bread, Refrigerator, are tools, objects or products that one may use or consume daily.

## Experiment materials, and methods

As described in the introduction section, the main hypothesis of this paper was to compare the learning using the selected EPM-based Learning Content (ELC) with the TLC. An experiment was conducted with the non-literate population to validate this hypothesis. This section presents the details of the experiment. Firstly, the demographics of the participants are detailed. Secondly, the material used in the experiment, and the procedures are discussed.

### Participants

Initially, 121 participants with no literacy were selected, however, were reduced to 107 participants after the scrutiny. In the scrutiny, participants’ age was determined along with their skills to pronounce and recognize alphabet letters of Urdu language. Only the adults with age greater than 15 and with no skills to pronounce and recognize alphabet letters were selected. Out of 107 participants, 60 were male, and 47 participants were female. Most of them were practicing jobs like the female worker who babysit, the male workers doing jobs in the offices requiring no literacy etc. Only 10 of the selected participants had attended up to 1.5 years of formal schooling. Only 5 of the participants had a computer at home, however, none of them ever used it. All participants had seen a computer and they were motivated to get an education through computers.

### Procedure

The experiment followed a between-subject design, where the participants were randomly divided into two groups, i.e., the experimental group (EG) and the control group (CG). The EG (54 participants) was presented with ELC and the CG (53 participants) was presented with TLC. This was materialized by developing two CAL applications for the two groups. One of the application supported alphabet letter learning using EPM, while the other used TLC. Fig 1 illustrates the user interface of the CAL application using ELC. The CAL applications were designed to minimize the role of instructors and to reduce the cognitive load in addition to giving immediate feedback [28].

**Fig 2:**
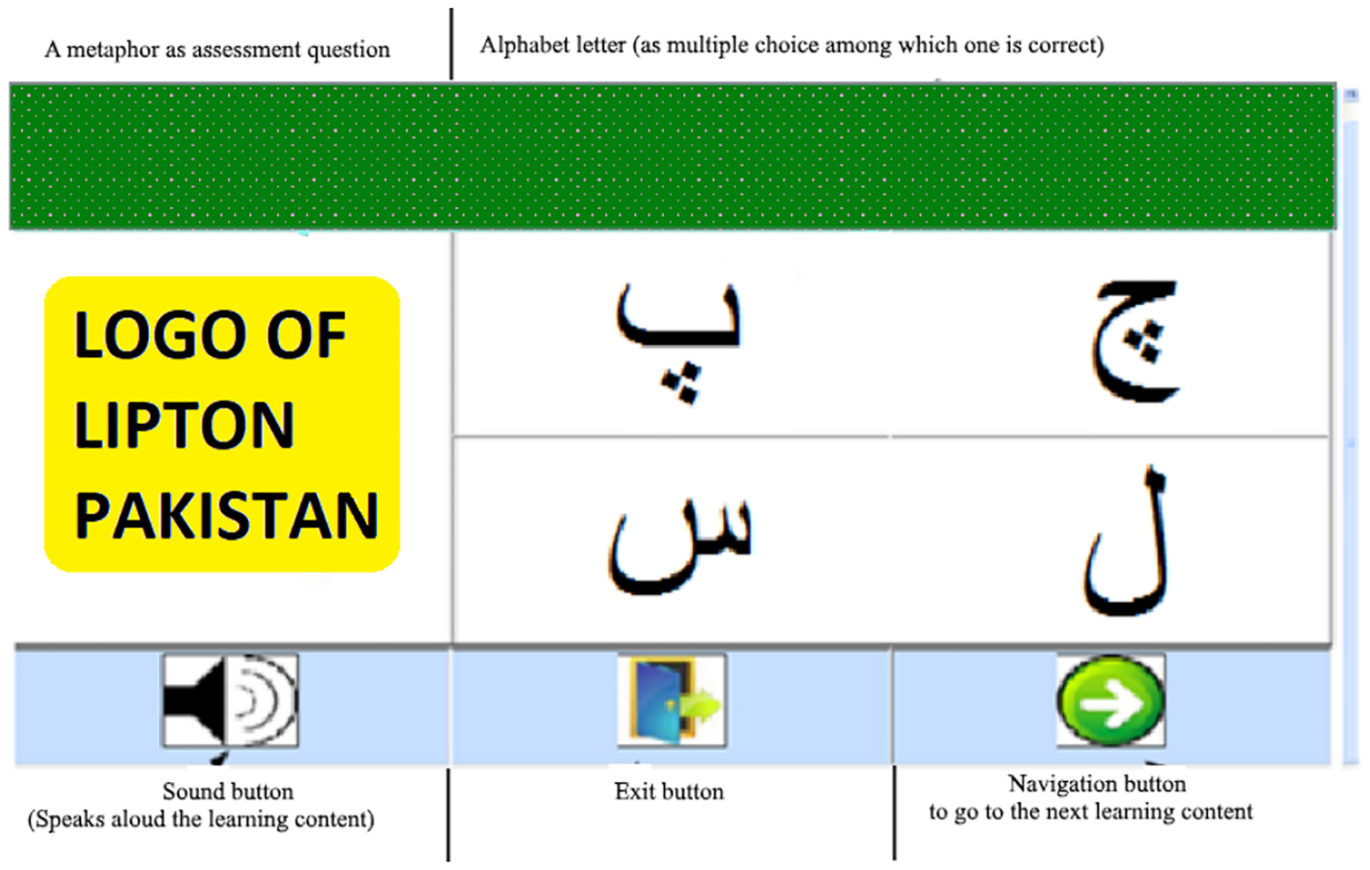
Computer-assisted assessment application using EPM.

The experimental procedure was as follows: A demo on the use of the applications’ interface was given to the participants for 30 minutes before starting the learning session. The learning sessions continued for one hour a day on the weekdays (excluding the weekends) for four weeks. It was made sure that the participants should learn all the alphabet letters on a given day. After completion of the sessions, two assessment tests were conducted: a computer-assisted assessment for recognition of alphabet letters and a manual assessment of the correctness of alphabet letters pronunciations. For computer-assisted assessment, another application was developed. The user interface of this application is illustrated in Fig 2. During the computer-assisted assessment, a picture was presented to the learner, and he/she was required to select the correct alphabet letter representing the picture by clicking on it via mouse (c.f. Fig 2). For the CG, the ELC was not included. One of the authors of this paper was present in the assessment session to help participants in case of problems like turning-on computers, navigation through the environment or other unforeseen technical issues. At the end of the assessment of both groups were informed about their mistakes via signs of ticks or crosses against each correct and incorrect answer. During the manual assessment, an alphabet letter along with the EPM content was presented to the EG. The CG was presented with alphabet letter and their corresponding image content. Learners from both groups were asked to pronounce the shown alphabet letter. The responses of both groups were noted manually in a text file against a unique participant id.

## Results and discussions

We started the analysis by conducting a two-way ANOVA. The Gender (Male vs. Female) and Group Type (the EG vs. CG) were taken as fixed factors and the recognition score as the independent variable. A similar analysis was again conducted for pronunciation scores. The results found no interaction effect for both the independent variables. Furthermore, a two-way ANOVA was also conducted by considering Group type and age group as fixed factors and recognition, pronunciation scores as independent variables. The results again revealed no significant interaction effect. In the scenario, various statistical experts such as Leech et al. [58] recommend examining and reporting the significant main effects one at a time. Therefore, we further conducted only the independent sample t-tests as explained in the following sections.

### Recognition

Recognition scores of the EG and the CG were analyzed. It was hypothesized that the learners in the EG would score higher than the learners in the CG. An independent t-test was conducted to compare the means of recognition scores of both the groups. The results showed that the mean score of the EG was significantly better than the mean score of the CG (t (54, 2) = 3.94, p < 0.001). The mean recognition score of the EG (μ = 20.50, SD = 8.489) was greater than the mean score of the CG (μ = 14.99, SD = 5.754). The results confirmed the hypothesis (H_1_) suggesting that the learners achieve higher recognition performance when presented with environmental print material as learning content.

To further discover the contributions of age and gender, the EG and the CG were divided into two age groups of age 15–40 (Younger) and 41–60 (older) as well as into two groups of gender male and female. The purpose of this division was to study the effect of the EPM on the learning of different age groups and gender groups, because of variant learning performance in different learning environments[11]. An independent t-test was conducted to compare the means of recognition scores of both age groups and gender groups separately. The results showed that the EG were significantly better than the CG with (t (46, 2) = 2.27, p = 0.028) and (t (61, 2) = 3.19, p = 0.002). The mean recognition score of the EG between age 15–40 was (μ = 20.00, SD = 8.63) greater than the mean score of the CG (μ = 15.09, SD = 5.74). Similarly mean recognition score of the EG between age 41–60 was (μ = 20.87, SD = 8.50) greater than the mean score of the CG (μ = 14.92, SD = 5.85).

Regarding male and female groups comparable results were found between experimental and the CG. The male the EG achieved significantly better recognition score than male the CG and female the EG achieved a significantly better recognition score as compared to female the CG with (t (60, 2) = 3.32, p = 0.002) and (t (47, 2) = 2.15, p = 0.037) respectively. The mean recognition score of male the EG was (μ = 20.98, SD = 8.34) greater than the mean score of male the CG (μ = 14.75, SD = 5.74). Similarly, the mean recognition score of female the EG was (μ = 19.98, SD = 8.78) greater than the mean score of the CG (μ = 15.35, SD = 5.88). The results suggest that the learners achieved higher recognition performance when presented with environmental print material as learning content. The further division of participants in age and gender groups did not change the results thus providing convincing evidence for acceptance of hypothesis H_1_. The results are summarized in Table 4 below.

**Table 4:** Statistics of alphabet letter recognition scores of the experimental and the CG.

Nature	Groups	N	Mean	SD	t-test for Equality of Means	
Recognition Score (Overall)	Experimental	54	20.500	8.489	t-value	p-value
	Control	53	14.990	5.754	3.936	<0.001
Recognition Score (Age 15–40)	Experimental	23	20.000	8.636	t-value	p-value
	Control	23	15.087	5.743	2.272	0.028
Recognition Score (Age 41–60)	Experimental	31	20.871	8.502	t-value	p-value
	Control	30	14.916	5.859	3.193	0.002
Recognition Score (Male)	Experimental	28	20.982	8.340	t-value	p-value
	Control	32	14.750	5.747	3.323	0.002
Recognition Score (Female)	Experimental	26	19.980	8.782	t-value	p-value
	Control	21	15.357	5.886	2.152	0.037

### Pronunciation

The pronunciation scores obtained from the participants of experimental and the CG were compared by conducting Independent t-tests. The results showed that the mean score of the EG was significantly better than the mean score of the CG (t (54, 2) = 3.86, p < 0.008). The mean pronunciation score of the EG was (μ = 18.03, SD = 7.95) greater than the mean score of the CG (μ = 12.85, SD = 5.81) thus suggesting that the learners achieved a better pronunciation score when assessed using the EPM-based content.

Pronunciation scores were also analyzed with respect to age and gender. Two age groups in the range [15–40] and [41–60] and two gender groups male and female were formed. Independent t-tests were conducted to compare the means of pronunciation scores of both age groups and gender groups separately. The results of younger vs. older age groups showed that the EG was significantly better than the CG with (t (46, 2) = 2.90, p = 0.006) in case of young participants and (t (61, 2) = 2.62, p = 0.011) in case of older participants. The mean pronunciation score of the EG [15–40] was (μ = 18.82, SD = 6.79) greater than the mean score of the CG (μ = 13.28, SD = 6.15). Similarly, the mean pronunciation score of the EG between age [41–60] was (μ = 17.54, SD = 8.77) greater than the mean score of the CG (μ = 12.51, SD = 5.62).

Regarding the male and females’, the EG did better as compared to the CG. The male EG achieved a significantly better pronunciation score than the male CG and the female EG achieved a significantly better pronunciation score as compared to the female CG with (t (60, 2) = 3.27, p = 0.002) and (t (47, 2) = 2.43, p = 0.02) respectively. The mean pronunciation score of the male EG was (μ = 19.18, SD = 7.11) greater than the mean score of the male CG (μ = 13.64, SD = 5.81). Similarly, the mean pronunciation score of the female EG was greater (μ = 16.81, SD = 8.75) than the mean score of the CG (μ = 11.64, SD = 5.75). The results suggest that the learners pronounced accurately when presented with EPM as learning content as compared to traditional content. The results, therefore, resulted in the acceptance of hypothesis H_2_. The results show that age and gender did not change the main results thus provided strong evidence of the effect of EPM on non-literate population pronunciation of alphabet letter. The results are summarized in Table 5 below.

**Table 5:** Statistics of alphabet letter pronunciation scores of experimental and the CG.

Nature	Groups	N	Mean	SD	t-test (Not Equal variances)	
Pronunciation Score (Overall)	Experimental	54	18.037	7.951	t-value	p-value
	Control	53	12.849	5.814	3.857	<0.001
Pronunciation Score (Age 15–40)	Experimental	23	18.826	6.791	t-value	p-value
	Control	23	13.282	6.154	2.901	0.006
Pronunciation Score (Age 41–60)	Experimental	31	17.451	8.777	t-value	p-value
	Control	30	12.516	5.623	2.623	0.01
Pronunciation Score (Male)	Experimental	28	19.178	7.105	t-value	p-value
	Control	32	13.640	5.807	3.276	0.002
Pronunciation Score (Female)	Experimental	26	16.807	8.745	t-value	p-value
	Control	21	11.642	5.753	2.430	0.019

### Recall rate

To find the recall score, the participants were informed on last day of an experiment to meet again after 6 weeks. Participants were reminded again a week before the meeting date. However, all the participants were not able to participate on the day. A total of 42 participants from the EG were present (total absent = 12, male = 5, female = 7) while in the CG only 27 participants were present (total absent = 26, males = 10, female = 16). These participants’ recognition and pronunciation scores were collected again using similar assessment setup as described in section 5.2. These scores will be called as recall scores in this section.

An independent t-test was conducted to find the differences in recall scores of both experimental and the CG. Significant differences of the recall (recognition (t (69, 2) = 5.18, p < = 0.0001) and pronunciation (t (69, 2) = 4.57, p < = 0.0001)) appeared as a result thus confirming hypothesis H_3_ as well. Furthermore, it was seen that the difference of the mean scores of recognition of the EG (μ = 18.2, SD = 8.01) and the CG (μ = 10.94, SD = 3.41) was lower than the difference of earlier mean recognition scores of the overall experimental (μ = 20.50, SD = 8.489) and the CG (μ = 14.99, SD = 5.754). Similarly, a difference of the mean scores of the pronunciations of the EG (μ = 16.45, SD = 7.84) and the CG (μ = 9.02, SD = 5.65) was lower than the difference of earlier mean pronunciation scores of the overall experimental (μ = 18.04, SD = 7.95) and the CG (μ = 12.84, SD = 5.81). The reason might be the time lapse of 6 weeks would have corroded the learning process as the participants did not practice the content again in the elapsed time.

No further analysis of the impact of age and gender were conducted because of availability of limited data. The acceptance of the three sub-hypotheses also confirmed the main hypothesis Hm that content based on EPM can enhance adult non-literate populations’ learning as compared to traditional content. The learning in this context was measured in terms of recognition, pronunciation, and recall rates. The results are summarized in Table 6 below.

**Table 6:** Statistics of alphabet letter recall scores of experimental and the CG.

Nature	Groups	N	Mean	SD	t-test (Not Equal variances)	
Recognition Recall Score	Experimental	42	18.20	8.014	t-value	p-value
	Control	27	10.94	3.414	5.183	0.000
Pronunciation Recall Score	Experimental	42	16.45	7.84	t-value	p-value
	Control	27	9.02	5.65	4.56	0.000

### Meta-analysis

In this study, we conducted 12 different analyses for recognition, pronunciation, and recall. The effect of EPM on participants learning was found significant in all the analyses as compared to the CG w.r.t age, gender and combined. However various researchers like Neyeloff et al. [59] highlighted that meta-analysis has various advantages over the testing statistical significance alone. For example, a meta-analysis could help in deciding the effectiveness of interventions as well help to answer beyond “Does it work or not” to “How does it work in the range of contexts”. It also promotes more scientific knowledge by adding more weight to the effect of size rather than statistical significance only. Therefore, we conducted a meta-analysis as follows.

**Fig 3:**
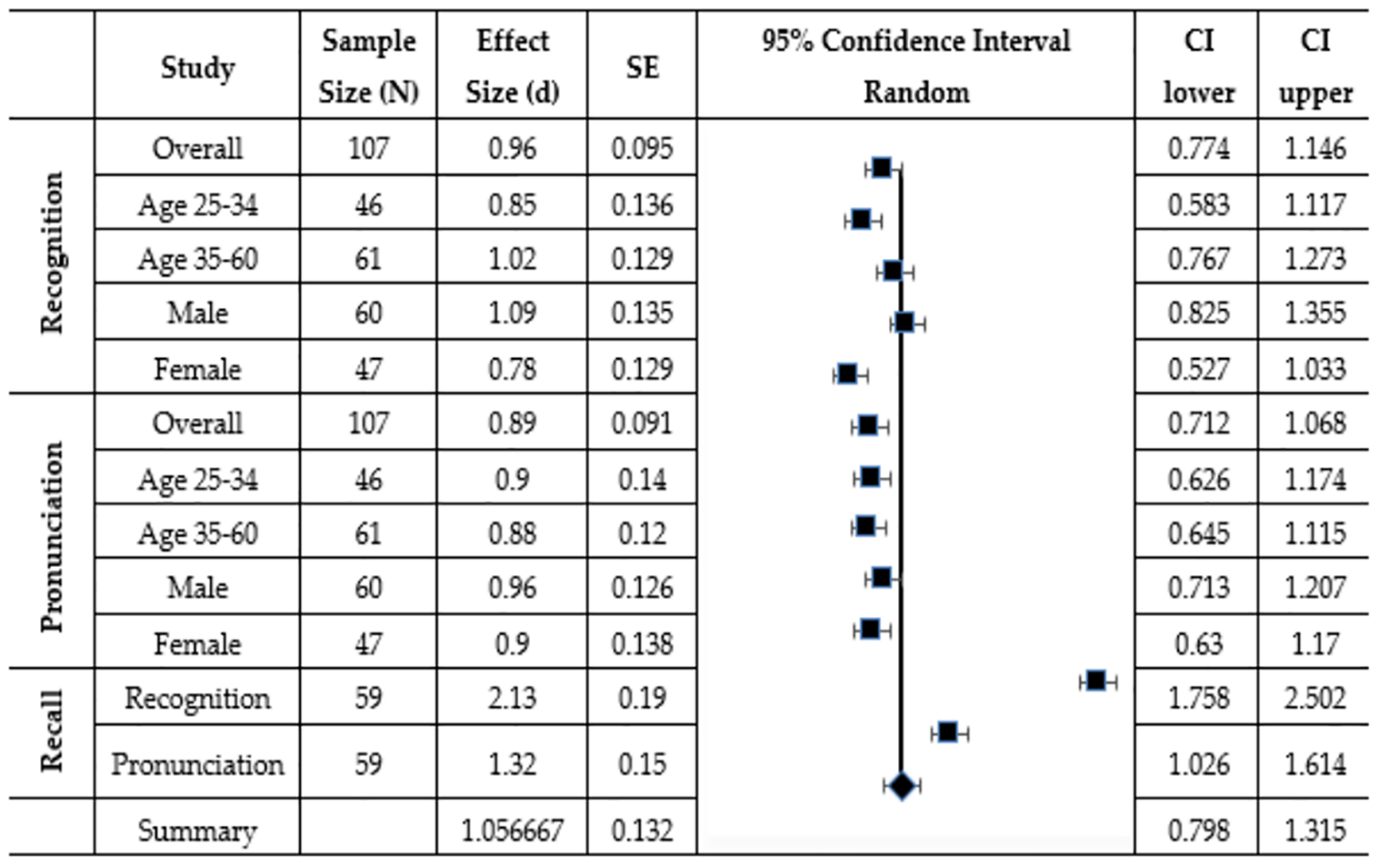
Meta–analysis table.

The first step in conducting a meta-analysis was the calculation of effect size for each analysis. The standard deviation was taken from a pooled value from both the EG and the CG and is favored by [59]. Similarly, Standard Error (SE) and random confidence intervals (CI) were calculated as well as a forest plot was created as discussed in [59]. According to [58] an effect size of 0.2 is small, an effect size of 0.5 is medium and an effect size of 0.8 or greater is large. According to the said classification, all the effect size was in the range (0.85–2.32) and therefore could be categorized as having a large effect (c.f. Fig 3). This means that in all the analyses, the participants who taught with the environmental print material (the EG) not only did better in recognition, pronunciation and recall significantly but also with bigger margins. This also is clear from the overall meta-analysis summary described in Fig 3 with a mean effect size of 1.05 (large) and with Confidence interval in the range (0.798–1.315). Fig 3 also explain the effect sizes of all the analyses with the help of a forest plot with the central tendency line standing for the combined effect size of all the analyses.

### Implications of the results

The studies found a significant effect of the ELC over TLC. A natural query could be that the effect could also be attributed to the age differences between the two groups. As the two groups were divided randomly, therefore, the age difference was not controlled. Finding the age effect on the learning outcomes could have important implications. To find the implications, the ages of the EG and CG were compared via an independent sample t-test. The test variables were the ages of the groups and the grouping variables were the type of the groups i.e. the EG and the CG. The results revealed no significant differences between the ages of the two groups (t = 0.16, p > 0.8). Therefore, any effect on the pronunciation, recognition, and recall scores thus could only be attributed to the content.

The selected ELC from the study-II were further compared with the TLC in terms of complexity of the content differences. Naturally, the more complex content could have affected the learning outcomes of the adult non-literate participants. Therefore, to match the materials, the number of letters and the number of syllables against each alphabet content of the ELC and TLC were extracted. A paired sample T-Test was conducted by taking a number of letters of the EPM-based content and the TLC. A similar analysis was again conducted with the number of syllables of ELC and TLC content. The analysis regarding a number of letters in the ELC vs TLC found that there are significantly more letters (t = 2.828, p < 0.01) in ELC as compared to the TLC (ELCμ = 4.16, TLCμ = 3.76). This further strengthens our hypothesis that EPM based content enhance the literacy skills of the adult non-literate population, despite being more complex and lengthier. However, there were no significant differences found between syllables of the ELC and TLC.

## Conclusions

The aim of the research was to study the impact of EPM-based learning content on the learning of the non-literate learners. To investigate this, two ethnographic studies were conducted to select the EPM-based content. The selected EPM-based learning content was then compared with TLC in an experiment with the adult non-literate population. The results of this experiment showed that the experimental group, (who have learned with the EPM-based learning content) outperformed the control group (who have learned with the traditional content) in terms of the learning parameters (recognition, pronunciation, recall). Hence the results confirmed that the carefully selected content relevant to the interests and demographics of the adult learners could enhance their learning.

As discussed in section 3, various approaches have studied the use of EPM content for the effective learning of the non-literate population [17, 18] and preschool children [19]. There has been a consensus that the interaction with the EPM content in socio-cultural context can be helpful in the learning of the alphabet letters. However, the non-literate users and the preschool children are not good at reading the EPM content out of context [17, 19]. In our experiment, we have presented the EPM content to the non-literate users in the context. Our results show that the users can recognize, pronounce and recall the alphabet letters in the EPM content. Therefore, the results are aligned with the previous work.

This study also has some limitations. One of them is related to the short duration of the learning sessions as compared to the learning ABE programs, which span over the years. However, the learning content was also simple since the participants were adult learners. In addition, the sample size for such studies was not adequate considering a between-subject study. This is true specifically in case of finding the effect of EPM on recall in which half of the participants did not appear. However, some studies such as Khan et al. [8] were conducted on an even lesser number of participants. As a future work, the same study could be conducted with a larger sample size so that differences across genders and age groups may further be strengthened. Formal assessment of the intelligence of the participants could have helped to further generalize the results. Though the participants were randomly divided into two groups based on the assumptions of equal division of intelligence, yet this is an important prospect to investigate in the future studies. In addition, some of the non-literate users may pay more attention to the EPM as compared to the other non-literate users. This can hinder the chances for some non-attentive user to learn from EPM. While an analysis of the attention bias may be helpful in determining the applicability of our results in the wild, we did not gather such data. It is, therefore, a limitation of our study and a possible future direction. Furthermore, the learning content could be broadened from alphabet letters to forming words and beyond from the use of EPM. The impact of the EPM in the same context needs to be investigated further for rural and urban areas.

## Supporting information

S1 Table The result of the ethnographic studies-The selected EPM content. (DOCX)

S2 Table The Traditional Learning Content (TLC). (DOCX)

S1 File The results related data of the groups EG and CG. (SAV)

## References

[1] Cohen M, Wiek A, Kay B, Harlow J. Aligning public participation to stakeholders’ sustainability literacy—A case study on sustainable urban development in Phoenix, Arizona. Sustainability. 2015;7(7):8709–28.

[2] Statistics UIf. Adult and youth literacy. 2013.

[3] Admin. Balochistan with 43pc has the lowest literacy rate in Pakistan: Data Stories 2015. https://www.datastories.pk/balochistan-with-43pc-has-the-lowest-literacy-rate-in-pakistan/.

[4] Why Pakistan needs a literacy movement? UNESCO Islamabad, 2012.

[5] Hunt F. Dropping out from school: A cross-country review of the literature. Research Monograph No. 16. 2008.

[6] Teacher excellence and adult literacy: Just write a guide. 2012.

[7] Burmark L. Visual Literacy: Learn To See, See To Learn: ERIC; 2002.

[8] Khan IA, Hussain SS, Shah SZA, Iqbal T, Shafi M. Job search website for illiterate users of Pakistan. Telematics and Informatics. 2017;34(2):481–9.

[9] Aanstoos J, editor Visual literacy: an overview. Proceedings of the 32nd Applied Imagery Pattern Recognition Workshop; 2003: IEEE.

[10] Warschauer M, Liaw ML. Emerging Technologies in Adult Literacy and Language Education. National Institute for Literacy, 2010.

[11] Iqbal T. Investigating 3D Virtual World for Adult Basic Education (Ph.D. thesis) Vienna, Institute of Software Technology and Interactive Systems, Vienna University of Technology, Austria2012.

[12] Shamim A, Khan TA, Elahi M, Mohsin S. Mobile Based User-Centered Learning Environment for Adult Absolute Illiterates. Mobile Information Systems. 2016;2016.

[13] Eberle A, Robinson S. The adult illiterate speaks out: Personal perspectives on learning to read and write. Opinion Papers; Reports—Descriptive. Washington, DC.: National Institute for Community Development, 1980.

[14] Smith F. Learning to read: the never-ending debate. The Phi Delta Kappan. 1992;73(6):438–41.

[15] Park JH, Choi HJ. Factors influencing adult learners’ decision to drop out or persist in online learning. Educational Technology & Society. 2009;12(4):207–17.

[16] Lave J, Wenger E. Situated learning: Legitimate peripheral participation: Cambridge University Press; 1991.

[17] Kurvers J, van Hout R, Vallen T. Print awareness of adult illiterates: a comparison with young pre-readers and low-educated adult readers. Read Writ. 2009;22:863–87. 10.1007/s11145-008-9129-7 19768120 PMC2744801

[18] Neumann MM, Hood M, Ford RM, Neumann DL. The role of environmental print in emergent literacy. Journal of Early Childhood Literacy. 2012;12(3):231–58.

[19] Cardoso-Martins C, Rodrigues LA, Ehri LC. Place of environmental print in reading development: evidence from nonliterate adults. Scientific Studies of Reading. 2003;7(4):335–55.

[20] Schlechty PC. Shaking up the schoolhouse how to support and sustain educational innovation. Wiley, 2004.

[21] Education indicators: Technical guidelines. 2009.

[22] Wagner DA. IT and education for the poorest of the poor: Constraints, possibilities, and principles. TechKnowlogia: International Journal for the Advancement of Knowledge and Learning. 2001.

[23] Rosen DJ. Using electronic technology in adult literacy education In: Comings J, Garner B, Smith C, editors. The annual review of adult learning and literacy. 1: Jossey-Bass; 1999 p. 304–15.

[24] Wagner DA, Hopey C. Literacy, electronic networking and the internet. Technical Report: TR98-10. Philadelphia: the University of Pennsylvania, National Center on Adult Literacy, 1998.

[25] Sabatini JP. Designing multimedia learning systems for adult learners: Basic skills with a workforce emphasis. Philadelphia, PA: National Center on Adult Literacy, 2001.

[26] Askov EN, Johnston J, Petty LI, Young SJ. Expanding access to adult literacy with online distance education. Boston, MA: National Center for the Study of Adult Learning and Literacy, 2003.

[27] Stites R. Implications of new learning technologies for adult literacy and learning In: Comings J, Garner B, Smith C, editors. Review of adult learning and literacy. 4: Routledge; 2004 p. 109–56.

[28] Wagner DA, Kozma RB. New technologies for literacy and adult education:. Paris: UNESCO; 2005.

[29] APPEAL training materials for literacy personnel. 1988.

[30] Chall J. Learning to read: The great debate 1967.

[31] Goodman K. What’s whole in whole language: 20th anniversary edition: RDR Books; 2005.

[32] Onyia MN. Instructional materials and design: Issues and challenges. Academic Journal of Interdisciplinary Studies. 2013;2(6):153.

[33] Kress G, van Leeuwen T. Multimodal discourse: The modes and media of contemporary communication: Hodder Education, UK; 2001.

[34] Mayer RE. Multimedia learning: Cambridge University Press; 2001.

[35] Knowles MS. The adult learner: A neglected species (building blocks of human potential): Gulf Publishing Co.; 1990.

[36] Smith L. Necessary knowledge: Piagetian perspectives on constructivism: Psychology Press; 1993.

[37] Pritchard A. Ways of learning: Learning theories and learning styles in the classroom: Routledge; 2009.

[38] Selinger M. Setting authentic tasks using the internet in schools In: Leask M, editor. Issues in teaching using ICT: Routledge; 2001.

[39] Keller JM. Motivational design of instruction. Instructional Design Theories and Models: An Overview of their Current Status. 1983;1:383–434.

[40] Felder RM. Matters of style. ASEE Prism. 1996;6(4):18–23.

[41] Khan FA, Graf S, Weippl ER, Tjoa AM, editors. An approach for identifying affective states through behavioral patterns in web-based learning management systems. in Proceedings of the 11th International Conference on Information Integration and Web-based Applications & Services; 2009.

[42] Tortorella RA, Graf S. Considering learning styles and context-awareness for mobile adaptive learning. Education and Information Technologies. 2017;22(1):297–315.

[43] Kolb DA. Experiential learning: Experience as the source of learning and development: Prentice-Hall, Inc; 1984.

[44] Miller D. Executive summary of the use of educational software in adult literacy programs: A comparison of integrated learning systems and stand-alone software. Information Analyses; Reports—Evaluative. Ottawa (Ontario), Canada: National Literacy Secretariat, 1996.

[45] McCambridge TR. Drill and practice. California Lutheran University 2017.

[46] Skinner BF. Science and human behavior. New York: The Free Press; 1953.

[47] Sleeter CE, Grant CA. Race, class, gender, and disability in current textbooks In: Apple MW, Christian-Smith LK, editors. The Politics of the Textbook 1991 p. 99.

[48] Prevedel A. The world view behind curriculum. Focus on basics. Focus on Basics. 2003;6(C):8–13.

[49] Smith C, Hofer J, Gillespie M. The working conditions of adult literacy. Focus on Basics. 2001:1–7.

[50] Kassow DZ. Environmental print awareness in young children. Seattle WA: Talaris Research Institute, 2006.

[51] McGee LM. Young children’s environmental print reading. Childhood Education. 1986;63(2):118–25.

[52] Ehri LC. Critique of five studies related to letter name knowledge and learning to read Reading research revisited: Merrill Pub Co; 1983.

[53] Whitehurst GJ, Lonigan CJ. Child development and emergent literacy. Child Development. 1998;69(3):848–72. 9680688

[54] Kuby P, Aldridge J. The impact of environmental print instruction on early reading ability. Journal of Instructional Psychology. 2004;31(2):106–14.

[55] Ahmad S. Pehla Qadam (First Step). Islamabad, Pakistan: Pakistan Academy of Social Sciences, PLCC and UNICEF, 1995.

[56] EEF. Khawandgi (Literacy) Project. Peshawar, Pakistan: Elementary Education Foundation (EFA), KPK, 2007.

[57] Nabi R. ESRA Parhai (reading). Islamabad, Pakistan: USAID, Pakistan, 2006.

[58] Leech NL, Barrett KC, Morgan GA. IBM SPSS for intermediate statistics: Use and interpretation: Routledge; 2014.

[59] Neyeloff JL, Fuchs SC, Moreira LB. Meta-analyses and Forest plots using a Microsoft Excel spreadsheet: step-by-step guide focusing on descriptive data analysis. BMC research notes. 2012;5(1):52. 22264277 10.1186/1756-0500-5-52 PMC3296675

