# Supplementary material for: Fighting adult illiteracy with the help of the environmental print material
Source: PLoS One. 2018 Aug 23;13(8):e0201902. doi: 10.1371/journal.pone.0201902 (PMC6107138; doi:10.1371/journal.pone.0201902)
Supplement: S1 Table — (DOCX) [file pone.0201902.s001.docx]

**S1 Table: The result of the ethnographic studies-The selected EPM content**. (Note: Due to possible copyrights and because of non-availability of the images under Creative Commons Attribution License (CCAL) CC BY 4.0, all the images are explained in textual form instead of providing original images)

| **Sr. No** | **Alphabet Letters** | | **Selected items** | | **Most frequently recognized item (percentage)** | **Images** |
| --- | --- | --- | --- | --- | --- | --- |
|  | **Standard Urdu Script** | **Roman Equivalent** | **Urdu Voice (Script)** | **Definition** |  |  |
| 1 | 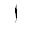 | Alaf | Akhbar  (اخبار) | Newspaper | 100 | **Image of a local news paper** |
| 2 | 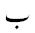 | Bay | Bata  (با ٹا) | Shoes Brand | 79 | **Logo of BATA** |
| 3 | 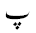 | Pay | Pepsi  (پیپسی) | Drink Brand | 90 | **Logo of PEPSI** |
| 4 | 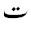 | Tay | Teer  (تیر) | Election symbol | 100 | **Image of an Arrow (Election Symbol)** |
| 5 | 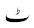 | Tay | Taxi  (ٹیکسی) | Vehicle | 76 | **Image of local Taxi** |
| 6 | 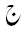 | Jeem | Geo  (جیو) | TV channel | 75 | **Image of GEO TV** |
| 7 | 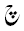 | Chay | Chingchi  (چنگچی) | Vehicle | 97 | **Image of a Tri wheeler passenger vehicle** |
| 8 | 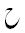 | Hay | Huqa  (حکا) | Smoking tool | 99 | **Image of a local smoking tool** |
| 9 | 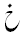 | Khay | Khargosh  (خرگوش) | Rabbit | 100 | **Image of a Rabbit** |
| 10 | 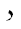 | Daal | Daig  (دیگ) | Cooking pot | 100 | **Image of big cooking pot** |
| 11 | 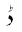 | Ddaal | Dalda  (ڈالڈا) | Cooking Oil Brand | 100 | **Logo of DALDA** |
| 12 | 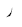 | Ray | Roti  (روٹی) | Bread (Chapati) | 100 | **Image of a loaf (Bread)** |
| 13 | 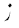 | Zay | Zong  (زونگ) | Mobile Service | 95 | **Logo of ZONG (Local mobile company)** |
| 14 | 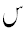 | Seen | Surf  (سرف) | Detergent Powder | 88 | **Image of the pack of a local detergent powder** |
| 15 | 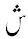 | Sheen | Shair  (شیر) | Election Symbol | 100 | **Image of a Lion** |
| 16 | 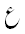 | Ain | Ainak  (عینک) | Spectacles | 100 | **Image of Spectacles** |
| 17 | 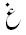 | Ghain | Ghobara  (غبارہ) | Balloon | 100 | **Image of Balloons tied together** |
| 18 | 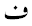 | Fay | Farij  (فریج) | Refrigerator | 100 | **Image of a opened refrigerator** |
| 19 | 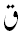 | Qaf | Quraan  (قرآن) | Holy book | 100 | **Image of the Holy book** |
| 20 | 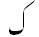 | Kaf | Camera  (کیمرہ) | Camera | 85 | **Image of a Camera** |
| 21 | 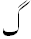 | Gaf | Goli  (گولی) | Tablet | 100 | **Image of the Tablets** |
| 22 | 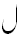 | Laam | Lipton  (لپٹن) | Tea Brand | 94 | **Logo of the LIPTON** |
| 23 | 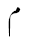 | Meem | Masjid  (مسجد) | Mosque | 100 | **Image of a Mosque** |
| 24 | 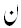 | Noon | Nokia  (نوکیا) | Cell phone brand | 99 | **Image of the Nokia 3310. A locally famous mobile phone** |
| 25 | 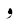 | Wow | Warid  (وارد) | Mobile Service | 92 | **Logo of WARID (A local mobile company)** |
| 26 | 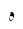 | Hey | Haaki  (ہاکی) | Hockey | 85 | **Image of a Hockey stick (Locally famous sport)** |
| 27 | 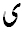 | Yay | Ufone  (یوفون) | Mobile Service | 89 | **LOGO of the Ufone (a local mobile company)** |
| 28 | 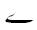 | Yay | Ufone  (یوفون) | Mobile Service | 89 | **LOGO of the Ufone (a local mobile company)** |
