# Supplementary material for: Fighting adult illiteracy with the help of the environmental print material
Source: PLoS One. 2018 Aug 23;13(8):e0201902. doi: 10.1371/journal.pone.0201902 (PMC6107138; doi:10.1371/journal.pone.0201902)
Supplement: S2 Table — (DOCX) [file pone.0201902.s002.docx]

**S2 Table: The Traditional Learning Content (TLC)** (Note: Due to possible copyrights and because of non-availability of the images under Creative Commons Attribution License (CCAL) CC BY 4.0, all the images are explained in textual form instead of providing original images)

| **Sr. No** | **Alphabet Letters** | | **Selected items** | | **Images** |
| --- | --- | --- | --- | --- | --- |
|  | **Standard Urdu Script** | **Roman Equivalent** | **Urdu Voice (Urdu Script)** | **Definition** |  |
| 1 | 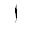 | Alaf | Anar  (انار) | Pomegranate | **Image of a Pomegranate** |
| 2 | 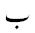 | Bay | Bakri  (بکری) | Goat | **Image of a Goat** |
| 3 | 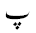 | Pay | Pankha  (پنکھا) | Drink Brand | **Image of a Pedestal Fan** |
| 4 | 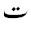 | Tay | Taaj  (تاج) | Crown | **Image of a Crown** |
| 5 | 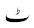 | Tay | Tokri  (ٹوکری) | Basket | **Image of a Basket** |
| 6 | 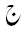 | Jeem | Joota  (جوتا) | Shoe | **Image of a Shoe** |
| 7 | 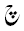 | Chay | Chaaqoo  (چاقو) | Knife | **Image of a Knife** |
| 8 | 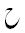 | Hay | Halwa  (حلوہ) | Sweet Dish | **Image of a Sweet Dish** |
| 9 | 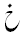 | Khay | Khaala  (خالہ) | Aunt | **Image of an Aunt (Head covered)** |
| 10 | 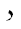 | Daal | Dawaat  (دوات) | Inkpot | **Image of an Inkpot** |
| 11 | 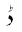 | Ddaal | Dakia  (ڈاکیا) | Postman | **Image of a Postman wearing local uniform** |
| 12 | 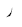 | Ray | Rasi  (رسی) | Rope | **Image of a Rope** |
| 13 | 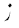 | Zay | Zuban  (زبان) | Tongue | **Image of a Human tongue** |
| 14 | 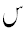 | Seen | Saag  (ساگ) | Vegetable (Spinach) | **Image of Spinach** |
| 15 | 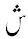 | Sheen | Shaakh  (شاخ) | Tree Branch | **Image of a Tree branch** |
| 16 | 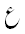 | Ain | Arbi  (عربی) | Arab | **Image of an Arab person** |
| 17 | 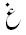 | Ghain | Ghaar  (غار) | Cave | **Image of a Cave** |
| 18 | 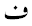 | Fay | Faraak  (فراک) | Dress | **Image of a Dress** |
| 19 | 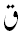 | Qaf | Qalam  (قلم) | Pen | **Image of a Pen** |
| 20 | 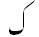 | Kaf | Kitaaab  (کتاب) | Book | **Image of a Book** |
| 21 | 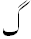 | Gaf | Gajjar  (گاجر) | Carrot | **Image of a Carrot** |
| 22 | 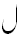 | Laam | Latto  (لٹو) | Yoyo | **Image of a YoYo** |
| 23 | 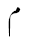 | Meem | Murghi  (مرغی) | Hen | **Image of a Hen** |
| 24 | 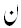 | Noon | Nalka  (نلکا) | Tap | **Image of a Tap** |
| 25 | 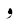 | Wow | Wazzoo  (وضو) | Ablution | **Image of a person doing ablution** |
| 26 | 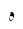 | Hey | Haathee  (ہاتھی) | Elephant | **Image of an Elephant** |
| 27 | 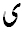 | Yay | Yakka  (یکہ) | Horse cart | **Image of a Horse Cart** |
| 28 | 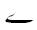 | Yay | Yakka  (یکہ) | Horse cart | **Image of a Horse Cart** |
